# Humor in Social Media Health Communication: A Systematic Review of Strategic Uses and Effects

**DOI:** 10.3390/bs16040509

**Published:** 2026-03-28

**Authors:** Yangna Hu, Cindy Sing Bik Ngai, Alex Chun Koon

**Affiliations:** 1 Department of Language Science and Technology, Faculty of Humanities, Hong Kong Polytechnic University, Hung Hom, Hong Kong SAR, China; yangna.hu@connect.polyu.hk; 2 School of Life Sciences, Faculty of Science, The Chinese University of Hong Kong, Shatin, Hong Kong SAR, China; alexkoon@cuhk.edu.hk

**Keywords:** humor, social media, health communication, communication strategy, communication effectiveness

## Abstract

Social media has become an important venue for health communication. Although prior research has examined the effects of humor, evidence on the mechanisms through which humor shapes communication effectiveness in social media health communication remains fragmented and has not been systematically synthesized. This systematic review examines how humor functions as a communication strategy in social media health communication designed by healthcare professionals, health organizations, and researchers. Following the Preferred Reporting Items for Systematic Reviews and Meta-Analyses (PRISMA) guidelines, 32 empirical studies were identified and synthesized. Findings indicate that humor is primarily used in two ways: as a content-level strategy to enhance audience engagement and as a psychological persuasive appeal in health message design and dissemination. Across studies, humor not only enhanced platform-level engagement but also influenced affective responses, attitudes, cognitions, and perceptions, which in turn shaped health-related behavioral intentions. Importantly, the effectiveness of humor was also contingent upon contextual and audience characteristics. This review integrates fragmented evidence into a conceptual framework that clarifies the pathways and boundary conditions of humor-based health communication on social media. It also highlights key limitations associated with the use of humor in health messaging and outlines directions for future research. Overall, this study provides theoretical insights and practical guidance for the strategic use of humor in digital health communication.

## 1. Introduction

Humor is pervasively seen in advertising and commercial activities, which refers to the use of unexpected or incongruent elements that spark positive emotions while delivering a message (Apers et al., 2025; Skurka et al., 2018; Yoon & Tinkham, 2013). It is presented in various forms, such as sarcasm, satire, irony, and parody (Allem et al., 2023; Al-Rawi & Zemenchik, 2023). In addition to advertising, humor is widely applied to other fields, such as education, political communication, and management communication (e.g., Braha & Karabulut, 2024; Ngai et al., 2025; Penney, 2019). Humor is widely regarded as an effective persuasive communication strategy that operates through cognitive and affective pathways (Martin, 2007). It can capture attention, enhance motivation to process information, improve memory retention for message content (Cline & Kellaris, 2007; Eisend, 2009; Ittefaq et al., 2023), elicit laughter and positive emotions, which in turn enhance motivation to process information, improve memory retention and message acceptance (Eisend, 2009, 2011), and help diffuse tension in stressful situations (Warren et al., 2021).

In health communication, humor is employed by both individuals and healthcare organizations, albeit for different purposes. At the individual level, humor often serves as a coping strategy for patients living with chronic health conditions (Hilliard et al., 2015; Niela-Vilén et al., 2014), for nurses facing stress (Wanzer et al., 2005), and for people experiencing crises, such as during the COVID-19 pandemic, when humorous expressions helped alleviate fear and psychological stress (Al-Rawi & Zemenchik, 2023; Chibuwe & Munoriyarwa, 2022). On the other hand, healthcare organizations adopt humor strategically to engage the targeted audiences and facilitate message dissemination. Humor is frequently integrated into “infotainment” and “edutainment” formats to make health information more appealing, thereby increasing audience interest and promoting health-related intentions and behaviors (Galea et al., 2025; Heuss et al., 2023; McKee et al., 2004).

Specifically, humor-based strategies are widely used and studied in public health campaigns and in communication about stigmatized health issues. Within public health campaigns, humor is often positioned as an alternative to fear-based appeals and has been shown to effectively convey critical health information across various topics (Apers et al., 2025; So et al., 2016). Miller et al. (2021), for example, suggest that humor is widely used as a strategy in public health campaigns, such as those addressing binge drinking, breast and testicular cancer self-examination, and skin cancer, demonstrating its role in shaping health-related attitudes and behaviors, as well as encouraging interpersonal sharing that may indirectly influence health actions. Humor has also been found to be particularly valuable when communicating about stigmatized health issues, such as mental health issues and sexual health, where humorous framing can reduce stigma and increase willingness to share or engage with messages (Galea et al., 2025; Jones et al., 2014; Miller et al., 2021). Scholars argue that incorporating humor into sensitive health contexts may reduce fear, evoke positive emotional responses, and ultimately foster more receptive attitudes and adaptive health behaviors (Chan, 2014; S. Ford et al., 2021).

Social media has become an important venue for health communication (Ryan et al., 2022), offering unique opportunities to disseminate health messages and reach diverse audiences at relatively low cost (Gough et al., 2017; Kostygina et al., 2020). Prominent health agencies, such as the World Health Organization and the Centers for Disease Control and Prevention (CDC), as well as other health organizations, including hospitals, routinely use popular social media platforms to share health-related content, such as prevention guidelines, vaccination information, and research updates, often achieving substantial reach. For example, Heuss et al. (2023) analyzed 2326 social media posts from 27 Swiss hospitals during the early outbreak of COVID-19 and found that hospitals used social media, primarily Facebook and Twitter, more frequently than usual to communicate with the public. These organizations engaged audiences by disseminating COVID-related information, calling for action and solidarity, and reassuring the public, resulting in a subset of the posts (n = 37) going viral and generating extensive discussion in the comment sections. Moreover, the sharing affordances of social media platforms further amplify the reach of health information, particularly among young users who are more likely to share the links of health-related content to support family members and educate peers (McKee et al., 2004; Sharif et al., 2021).

Humor has been identified as a critical factor influencing audience engagement with health-related content on social media (Goodyear & Quennerstedt, 2020; Metzler et al., 2022; Piedra & Varea, 2023). For instance, Munawar and Prabhu (2021) found that humorous posts were among the most prevalent types in radiology-related discussions on Reddit, highlighting humor’s potential to enhance visibility in social media-based health communication. Similarly, Munro et al. (2024), in a cross-sectional content analysis of 250 TikTok videos related to dieting, reported that humor was significantly associated with higher levels of audience engagement, including the amount of likes, comments, and shares. Recently, researchers have increasingly examined memes, which are images, texts, GIFs, or videos that encapsulate ideas or catchphrases (Allem et al., 2023; Wu et al., 2025), as a dominant form of humorous expression in digital culture. Memes have become a key vehicle through which health organizations disseminate health messages and social media users replicate and circulate health-related content, contributing to the viral spread of health information on platforms like TikTok (Oh et al., 2023; Southerton, 2021; Wu et al., 2025; Zulli & Zulli, 2022). Taken together, these findings suggest that humor functions as an effective and widely adopted communication strategy in social media health communication (Nabi, 2016).

On the other hand, scholars have raised concerns about the effectiveness of humor in social media health communication, suggesting that its impact may not be connected with higher levels of audience engagement or even be counterproductive in certain situations (Galea et al., 2025; Kessel et al., 2016; Paek et al., 2013). For instance, Paek et al. (2013) used content analysis to study user comments on anti-smoking video posts on YouTube and found that humorous content actually decreased the likelihood of favorable message-oriented and audience-generated thoughts. Kessel et al. (2016) conducted focus groups with 19 Australian teenage girls about the feasibility and design of a social media health intervention for physical activity. Participants in this study reported that using humorous messages to present written messages in health interventions was “uncool,” showing that message senders were “trying hard.” In designing persuasive messages, the use of humor is debated, as it might trivialize the significance of health topics, such as vaccine injection (Oh et al., 2023).

Despite the growing body of empirical research examining humor in digital health contexts, there remains a notable lack of review studies that systematically synthesize how humor functions as a communication strategy in social media health communication, particularly with respect to the underlying mechanisms through which humor influences message effectiveness (Galea et al., 2025). Existing studies have largely focused on descriptive engagement metrics, such as likes, comments, and shares, leaving open the question of what other communication outcomes, such as changes in health-related perceptions, humor may produce in social media environments. Moreover, prior research often examines humor created or circulated by general users (e.g., Allem et al., 2023; Basch et al., 2021; Cottingham & Rose, 2023), which may primarily serve entertainment or coping purposes rather than deliberate health communication goals. By concentrating on professionally or institutionally generated content, we can examine humor as an intentional communication strategy rather than a spontaneous or user-driven expression.

To address the gap of examining humor as a communication strategy in social media health communication, this study aims to conduct a systematic review focusing on examining empirical evidence on the mechanisms through which humor operates as a communication strategy in social media-based health communication designed or informed by professionals and institutions. In particular, this review seeks to integrate existing evidence to clarify how humor influences communication effectiveness and to make sense of the mixed findings regarding the positive and negative effects of humor in social media health communication. Based on this objective, we propose the following research questions to guide this review:

RQ1: How is humor used as a communication strategy in social media health communication by professionals?

RQ2: What communication outcomes does humor produce in health-related messaging within social media environments?

RQ3: What are the empirically supported relationships between humor-based strategies and communication outcomes in social media health communication?

By integrating findings from empirical evidence, this review consolidates a fragmented body of literature and develops a coherent conceptual framework that clarifies the conditions under which humor enhances or undermines health communication effectiveness on social media. Practically, the findings provide evidence-based guidance for healthcare professionals, health organizations, health message designers and communicators, health policymakers, and researchers seeking to design and disseminate effective humor-based health messages, highlighting the conditions under which humor can provide desired health outcomes while avoiding potential pitfalls. In doing so, this study informs the strategic use of humor in social media health communication, discusses challenges of using humor, and identifies directions for future research aiming at improving public health communication in digital environments.

## 2. Methods

### 2.1. Search Strategy

This systematic review was conducted following the Preferred Reporting Items for Systematic Reviews and Meta-Analyses (PRISMA) guidelines (Page et al., 2021). We registered this systematic review on Open Science Framework (registration DOI is 10.17605/OSF.IO/9NQ23). We searched three databases, including Web of Science, PubMed, and Scopus, which index a wide range of peer-reviewed literature in healthcare, medical studies, and social sciences (S. Chen et al., 2024; Hu et al., 2025; W. Lu et al., 2024). To identify relevant studies, we employed three groups of search terms. The first group captured humor-related concepts (humor* OR laugh* OR joke* OR funny). The second group targeted health communication and included the keyword “health” to ensure a broad coverage of studies in this domain. The third group focused on social media contexts (“social media” OR “social network*” OR “social platform*”). Searches were conducted within the title and abstract fields of each database.

### 2.2. Eligibility Criteria

Studies were selected for inclusion based on the following criteria: (1) the study focused on health communication within social media contexts; (2) the study examined humor as a deliberate communication strategy used, or intended for use, in health-related settings, such as public health campaigns, health education, disease prevention, or stigma reduction; (3) the study featured message senders or designers who were healthcare professionals, health educators, health organizations, marketing agencies, researchers, or other formal organizations, rather than lay users; (4) the study reported or analyzed communication outcomes, such as audience engagement, message retention, attitudes, behavioral intentions, or other health-related outcomes. In addition, eligible studies were required to (5) employ empirical research methods, (6) be published as peer-reviewed journal articles, and (7) be written in English with full-text availability.

Studies were excluded if they did not address humor in health communication or examined humor exclusively in non-social media or traditional media contexts. Articles were also excluded if humor was not designed or used as a communication strategy, or if the humorous content was created solely by lay users for entertainment or social commentary. Furthermore, studies that did not report or discuss communication effectiveness outcomes, as well as non-peer-reviewed publications or articles without full-text availability in English, were excluded.

### 2.3. Study Selection

Three authors participated in the study selection process. After removing duplicates, the first reviewer (YH) and second reviewer (ACK) independently screened all titles and abstracts to assess eligibility. Discrepancies in eligibility judgments were resolved through discussion, with a third reviewer (CSBN) acting as an adjudicator where consensus could not be reached. During the full-text screening stage, the first and second reviewers (YH and ACK) independently evaluated all remaining articles, and any disagreements were resolved through consultation with the third reviewer (CSBN), who made the final determination.

**Figure 1:**
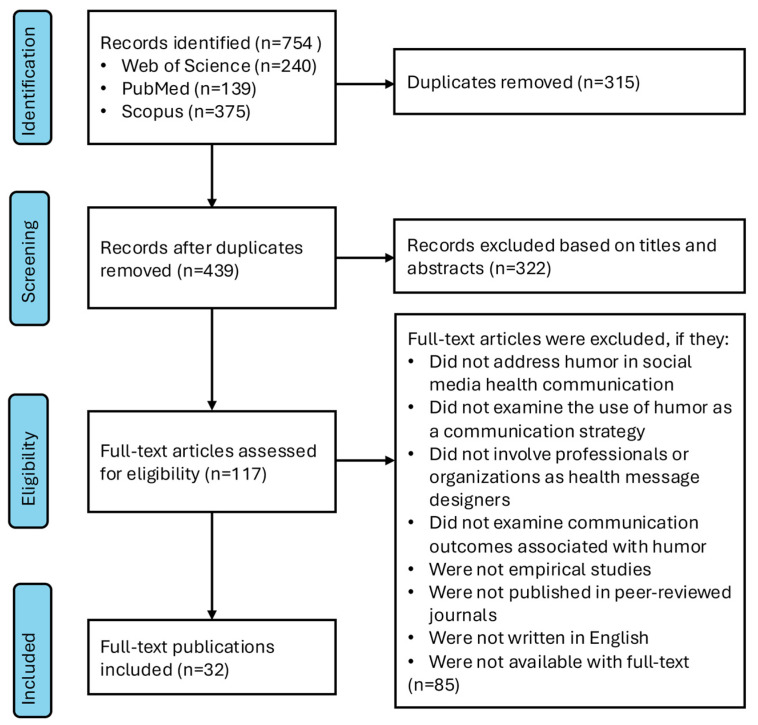
Literature screening process.

The initial database research was conducted on 2 October 2025, yielding a total of 754 records, including 240 studies from Web of Science, 139 studies from PubMed, and 375 studies from Scopus. These studies were published between 2000 and 2025. After removing 315 duplicate records, 439 studies remained for title and abstract screening based on the predefined inclusion and exclusion criteria. Following this screening, 322 studies were excluded, and 117 studies remained for full-text review. After full-text assessment, 32 studies were identified as meeting all inclusion criteria and were included in the final analysis, as they focused on humor as a health communication strategy within social media contexts. The included studies were published between 2013 and 2025. The study selection process is illustrated in Figure 1.

### 2.4. Quality Appraisal

We used the quality appraisal tool, Mixed Methods Appraisal Tool (MMAT; Hong et al., 2018), to examine the methodological quality of included studies. The MMAT provides a structured framework for appraising studies employing diverse research designs, including qualitative, quantitative, and mixed-method approaches. Following quality appraisal, the 32 included studies for this review demonstrated strong methodological qualities according to the MMAT criteria. Detailed appraisal results are reported in Table A1 of Appendix A.

### 2.5. Data Synthesis

Guided by the research questions, the analysis focused on three overarching themes: (1) how humor is employed as a communication strategy in health communication within social media contexts; (2) the communication outcomes associated with the use of humor in social media health communication; and (3) the empirically supported relationships between humor-based strategies and communication outcomes. The first reviewer (YH) independently extracted and coded all data relevant to these themes. An inductive coding approach was employed to allow themes and sub-themes to emerge from the data in response to the research questions (Naeem et al., 2023). To ensure reliability, the second reviewer (ACK) independently examined 18.75% of the coded studies. Any discrepancies or disagreements were resolved through discussion, with the third reviewer (CSBN) acting as an arbiter when consensus could not be reached.

A meta-analysis was not conducted because the included studies exhibited substantial methodological and contextual heterogeneity. Meta-analysis requires a sufficient level of homogeneity in study design, populations, interventions, and outcome measures to allow meaningful comparison of effect estimates (Borenstein et al., 2009). The included studies varied widely in health topics, research methods, participant groups, and social media platforms, and only a limited number reported comparable quantitative findings. Therefore, this systematic review did not statistically pool outcomes across studies; instead, a narrative synthesis was adopted to integrate the findings (Hu et al., 2026). This synthesis focused on identifying thematic patterns in how humor was used as a communication strategy, the communication outcomes associated with humor-based strategies, and the relationships between humor strategies and communication outcomes in social media health communication.

## 3. Results

The 32 included studies examined the mechanism through which humor operates as a communication strategy in health communication within social media contexts, spanning a wide range of health-related topics, such as mental health (Yap et al., 2019), antitobacco communication (Lee & Chen, 2017), HPV vaccination misinformation correction (Kim et al., 2021; Vraga et al., 2019), skin cancer prevention (Gough et al., 2017), blood lead testing (Love et al., 2026), sexual health (Byron et al., 2013; A. Chen et al., 2025; Droog et al., 2025; Evers et al., 2013), willingness to buy health food (Reijnen et al., 2024), and so on. Twitter (now X) emerged as the most frequently examined platform for studying humor as a communication strategy, with 10 studies (31.25%) primarily focusing on this environment. Other social media platforms, including Facebook, Instagram, TikTok, YouTube, and WeChat, were also studied for disseminating humor-based health messages and examining communication effectiveness.

Across the included studies, humor was conceptualized in different ways: it was treated as a message frame intended to evoke joy or laughter (e.g., Gough et al., 2017; Ruco et al., 2022; Xiao & Yu, 2022), categorized as a thematic content category in which health information is presented in an amusing manner compared with other thematic strategies, such as educational content (e.g., Bian et al., 2021; O’Kane et al., 2022; Weeks et al., 2022), or conceptualized as an emotional appeal in public health messages, often contrasted with other emotional appeals, such as fear or hope (e.g., Alhabash et al., 2022; Vaala et al., 2022, 2025; Yap et al., 2019). Operationally, humor was identified through different methodological approaches, such as qualitative coding of social media content (e.g., Al-Rawi & Zemenchik, 2023; Bian et al., 2021; Yap et al., 2019), interviews or focus group discussions on message design (e.g., Byron et al., 2013; A. Chen et al., 2025; Galea et al., 2025), or graphics or textual elements embedded in social media health messages in surveys or experimental settings (e.g., Kim et al., 2021; Teoh et al., 2019; D. Wang et al., 2023). Section 3.1, Section 3.2 and Section 3.3 present a synthesis of findings on how humor was strategically employed by professionals or institutions as a communication strategy in social media health communication, the communication outcomes associated with its use, and the empirical relationships between humor-based strategies and communication outcomes. The data extraction table of the included studies is presented in Table A2 of Appendix B.

### 3.1. Humor as a Communication Strategy in Social Media Health Communication

#### 3.1.1. Using Humor as a Content-Level Communication Strategy for Audience Engagement

This review identified the use of humor as a communication strategy to attract audience engagement on social media as a prominent theme in the literature. A total of 19 included studies (59.38%) fell into this category. These studies employed a range of research methods, including content analysis (e.g., Al-Rawi & Zemenchik, 2023; Bian et al., 2021; Yap et al., 2019), interviews (e.g., Galea et al., 2025; Pfeiffer et al., 2014), focus groups (e.g., Byron et al., 2013; A. Chen et al., 2025; Ruco et al., 2022), observational approaches (e.g., Harper et al., 2024; Love et al., 2026; D. Wang et al., 2023), cross-sectional surveys (Teoh et al., 2019) and a quasi-experiment (Gough et al., 2017) to examine the association between humorous health-related messages and audience engagement. Across these studies, social media engagement metrics, such as likes, comments, and shares, were primarily used as the main indicators of communication effectiveness.

The majority of studies in this category (18 out of 19, 94.74%) reported that humorous health messages generated higher levels of audience engagement. Specifically, humor was found to capture audience attention by increasing exposure to health-related content, as reflected in higher viewing or play counts of social media posts (Al-Rawi & Zemenchik, 2023; Harper et al., 2024; Love et al., 2026; D. Wang et al., 2023). Qualitative studies further corroborated these findings, with participants indicating that humorous health messages were more likely to catch their eye in crowded social media environments (A. Chen et al., 2025; Galea et al., 2025; Ruco et al., 2022). Studies also reported that audiences preferred to use likes/hearts to show their engagement (Bian et al., 2021; Harper et al., 2024), and humorous health messages enhanced message reach by encouraging sharing behaviors (Al-Rawi & Zemenchik, 2023; A. Chen et al., 2025). For example, Al-Rawi and Zemenchik (2023) found that humorous COVID-19-related messages posted by healthcare workers received the highest number of shares compared with other content themes, such as educational content, misinformation correction, or expressions of appreciation for healthcare workers.

In contrast, Yap et al.’s (2019) study in this category identified a negative association between humor and audience engagement. The authors examined mental health messages related to depression and anxiety posted by 65 health organizations on YouTube, analyzing how eight different message appeals, including affiliation, hope, humor, heroism/success, ease/convenience, guilt/shame, sorrow, and fear, were associated with audience engagement outcomes. Their findings indicated that humor was negatively associated with the number of likes and showed no statistically significant relationship with comments or shares. This result may be attributable to the sensitive nature of the mental health topics examined, where humorous framing may be perceived as incongruent with audience expectations or the seriousness of the subject matter.

#### 3.1.2. Humor as a Psychological Persuasive Appeal in Social Media Health Message Design and Dissemination

In our study, a total of 13 included studies (40.63%) employed experimental designs to examine how humor operated as a psychological persuasive appeal in the design or dissemination of health messages on social media. In contrast to studies focusing primarily on audience engagement, this body of research investigated a broader range of communication effects associated with humor-based strategies. Specifically, humor was embedded within social media health messages to enhance persuasive outcomes related to misinformation correction, health behavior or healthy product promotion, and the prevention of unhealthy behaviors.

Regarding humor as a persuasive appeal for misinformation correction, three studies (Droog et al., 2025; Kim et al., 2021; Vraga et al., 2019) examined the use of humor as a corrective strategy for addressing health misinformation on social media, focusing on contraception and HPV vaccine misinformation topics. Droog et al. (2025) found that humorous corrections reduced misinformation believability in contraception, favorable attitudes toward natural contraception, and perceived believability of misinformation, reporting that humor was effective for debunking false beliefs. Similarly, Vraga et al. (2019) found that humor-based corrective messages effectively reduced misinformation credibility and strengthened participants’ resistance to HPV vaccine misinformation. Importantly, they also identified a moderating role of pre-existing attitudes toward the HPV vaccine. Participants’ agreement with the scientific consensus on the HPV vaccine, categorized as dismissive, undecided, or convinced, shaped the effectiveness of humor-based corrections, with individuals already convinced of vaccine safety responding most positively to humorous corrective messages.

There is only one published journal article (Kim et al., 2021) employing an eye-tracking approach to investigate the mediating role of visual attention in the effectiveness of humorous corrections. Their findings indicated that humor increased attention to both the misinformation text and the corrective image, which subsequently reduced the perceived credibility of misinformation and, in turn, lowered HPV vaccine misperceptions. Together, these studies provided empirical evidence supporting the potential effectiveness of humor-based strategies for correcting health misinformation on social media, while also highlighting the importance of audience characteristics in shaping their impact.

Regarding humor as a persuasive appeal for health promotion or prevention, ten studies (31.25%) focused on the use of humor as a persuasive appeal to promote health-related attitudes, perceptions, and behaviors or to prevent risky health behaviors. Among these, studies (Apers et al., 2025; T. Wang & Pavelko, 2023; Xiao & Yu, 2022) reported that humor-based appeals were more effective than non-humorous messages in achieving desired communication outcomes. Specifically, humorous health messages were found to be more effective in increasing intentions to seek health information (Apers et al., 2025), as well as offline engagement intentions, source likability, and risk perception related to social distancing in low-severity health crisis situations (Xiao & Yu, 2022). In addition, T. Wang and Pavelko (2023) revealed that humor-based appeals positively influenced attitudes toward preventive health behaviors (e.g., hand washing, mask wearing, and social distancing) and subsequent behavioral intentions through a mediated pathway involving source likability of health messages, with perceived efficacy moderating the strength of these effects.

Recent studies (Alhabash et al., 2022; Vaala et al., 2022, 2025) have also explicitly compared humor-based appeals with fear-based appeals in social media health communication compared with fear. One study (Alhabash et al., 2022), examining intentions to purchase medications through social media, found that humor-based appeals were more effective than fear-based appeals in reducing perceived threat to freedom and message-induced anger, enhancing favorability toward health messages, and shaping behavioral responses, while also reducing purchase intentions. Pre-existing risk perceptions moderated the relationship between persuasive appeals and behavioral intentions. Similarly, another study (Vaala et al., 2022) focusing on social distancing during the early stages of the COVID-19 pandemic found that humorous tweets from the CDC elicited lower levels of fear and guilt, which were associated with reduced perceptions of COVID-19 susceptibility and severity. Political ideology further moderated the effects of emotional appeals on social distancing perceptions. Along this line of thought, Vaala et al. (2025) identified additional moderators, including well-being orientation (individual vs. collective) and trust in the CDC, which shaped the relative effectiveness of humor versus fear appeals in influencing emotional responses to COVID-19.

Other studies in this category examined humor-based persuasion under varying message and contextual conditions. For example, Lee and Chen (2017) found that humorous antitobacco messages on Facebook were more effective when embedded within health-focused commenting environments, resulting in higher risk perceptions of smoking, less favorable attitudes toward smokers, and stronger intentions to avoid smoking. Reijnen et al. (2024) studied the importance of rhetorical characteristics in humorous messages and reported that humor-based healthy food promotion posts without offensive elements, such as references to the word “fat,” generated higher intentions to purchase healthy food. Seo et al. (2025) demonstrated that the effectiveness of humor depended on message sequencing and audience psychological antecedents. Using a multi-page social media post promoting vaccination, they found that presenting humor before risk information increased perceived vaccine effectiveness among individuals with high levels of vaccination calculation (an individual’s tendency to engage in the cost–benefit calculation of vaccination). Finally, T. Wang and Pavelko (2024) found that the effectiveness of humor varied by humor type and regulatory focus, with congruity humor being more effective when paired with prevention-focused messages and aggressive humor performing better when aligned with promotion-focused messages.

### 3.2. Communication Outcomes of Humor in Social Media Health Communication

Based on the reviewed studies, communication outcomes associated with humor-based health messages on social media were synthesized into four categories: attitudes, perceptions, behavioral intentions, and social media engagement behaviors. Five studies (15.63%) examined how humorous health messages influenced individuals’ health-related attitudes. These included attitudes toward the public health messages (Alhabash et al., 2022; T. Wang & Pavelko, 2023; Xiao & Yu, 2022), attitudes toward unhealthy behaviors, such as smoking (Lee & Chen, 2017), and attitudes toward preventive behavior (Droog et al., 2025; T. Wang & Pavelko, 2023). Alhabash et al. (2022) found that participants expressed more favorable evaluations of humorous health messages, whereas T. Wang and Pavelko (2023) and Xiao and Yu (2022) demonstrated that attitudinal effects of humor were contingent on perceived efficacy and crisis severity, respectively. Lee and Chen (2017) further showed that exposure to humorous antitobacco videos shaped attitudes toward smokers differently depending on platform and commenting context, with less favorable attitudes reported when humorous health messages were embedded in health-focused Facebook discussions.

Health-related perceptions constituted another major outcome category. Studies examined the influence of humor on health misperceptions (Droog et al., 2025; Kim et al., 2021; Vraga et al., 2019) and health risk perceptions (Droog et al., 2025; Lee & Chen, 2017; Vaala et al., 2022, 2025; Xiao & Yu, 2022). In addition, multiple studies assessed behavioral intentions resulting from exposure to humorous health messages. These included viral behavioral intentions, such as liking, sharing, commenting on, and recommending health messages (Alhabash et al., 2022; T. Wang & Pavelko, 2024; Xiao & Yu, 2022); information-seeking intentions (Apers et al., 2025); and intentions to engage in or avoid health-related behaviors, including purchasing medications through social media (Alhabash et al., 2022), avoiding smoking (Lee & Chen, 2017), purchasing healthy food (Reijnen et al., 2024), and COVID-19 vaccination intentions (Seo et al., 2025).

As noted earlier, studies examining humor as a content-level strategy for audience engagement primarily relied on platform-level engagement metrics, such as likes, shares, comments, and views, as indicators of communication effectiveness. A summary of all communication outcomes examined across the included studies is presented in the data extraction table (see Table A2).

### 3.3. Empirically Supported Relationships Between Humor and Communication Outcomes

**Figure 2:**
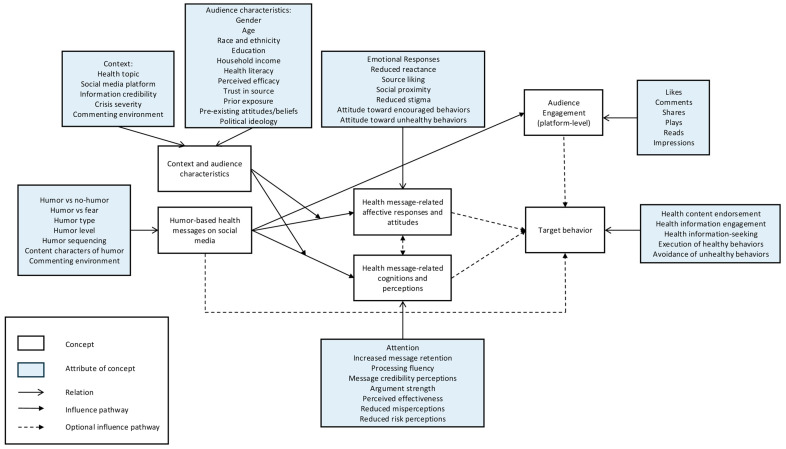
A conceptual framework of humor as a communication strategy employed by professionals in social media health communication.

To answer RQ3, we synthesized the empirically supported relationships between humor and communication outcomes in social media health communication and developed a conceptual framework grounded in the included studies (see Figure 2). The framework illustrates how humor-based health messages are connected with communication outcomes at the platform level (audience engagement outcomes) or operate through affective–attitudinal and cognitive–perceptual mechanisms, which might in turn shape communication outcomes at the individual level (attitudes, perceptions, and behavioral intentions). Importantly, these relationships are conditional, as they are shaped by contextual and audience-related characteristics.

#### 3.3.1. Direct Effects on Platform-Level Engagement

Across studies examining humor as a content-level strategy, empirical evidence consistently showed that humorous health messages generated higher platform-level engagement, including likes, shares, comments, views, and impressions (e.g., Al-Rawi & Zemenchik, 2023; Bian et al., 2021; Gough et al., 2017). These findings suggest a relatively robust direct relationship between humor and audience engagement, largely independent of downstream behavioral change. Humor appears particularly effective at increasing message visibility and circulation within social media ecosystems.

#### 3.3.2. Indirect Effects on Attitudes, Perceptions, and Behavioral Intentions

In contrast to audience engagement outcomes, direct effects of humor on target health behaviors were rarely observed. Only two studies reported direct behavioral outcomes associated with humor-based messages. Specifically, Apers et al. (2025) found that humorous health promotion messages increased intentions to seek additional health information, while Reijnen et al. (2024) showed that humor free of offensive language increased willingness to purchase healthy food. Beyond these limited cases, the majority of studies indicated that humor influenced behavioral intentions indirectly, operating through psychological and cognitive pathways.

As depicted in Figure 2, humor shaped communication effectiveness by influencing health message-related affective responses, attitudes, cognitions, and perceptions, which were examined in the reviewed studies both as proximal communication outcomes and as psychological mechanisms linking message design to downstream effects. At the affective-attitudinal level, humor was associated with increased source liking and social proximity (T. Wang & Pavelko, 2023, 2024), reduced fear, guilt, and psychological reactance (Alhabash et al., 2022; Vaala et al., 2022, 2025), and shifts in attitudes toward encouraged or discouraged behaviors (Lee & Chen, 2017). These affective–attitudinal responses functioned not only as outcomes but also as mediators, transmitting the effects of humor to behavioral intentions.

Similarly, humor also operated through cognitive–perceptual processes. Empirical evidence showed that humor increased visual attention (Kim et al., 2021), enhanced processing fluency (T. Wang & Pavelko, 2024), altered perceptions of message credibility and argument strength (Kim et al., 2021; Vraga et al., 2019), and reduced health-related misperceptions (Droog et al., 2025; Kim et al., 2021; Vraga et al., 2019). These cognitive and perceptual responses were analyzed as meaningful communication outcomes in their own right, reflecting how audiences interpreted and evaluated humorous health messages on social media. They were also conceptualized as mediating mechanisms, linking humorous messages and intentions to behavioral responses.

Importantly, evidence suggested potential trade-offs in these indirect pathways. Humor might weaken perceived argument strength or reduce perceived risk, particularly in high-severity contexts (Vaala et al., 2022; Xiao & Yu, 2022). Together, these findings support a mediated pathway whereby humor affects behavioral intentions primarily through changes in emotions, attitudes, cognitions, and perceptions rather than through direct persuasion.

#### 3.3.3. Moderating Role of Context and Audience Characteristics

Crucially, the framework highlights contextual and audience characteristics as key moderators that shape the effectiveness of humor-based health communication. Factors, such as crisis severity (Xiao & Yu, 2022), health topic sensitivity (Lee & Chen, 2017), platform affordances and commenting environments (Lee & Chen, 2017), perceived efficacy (T. Wang & Pavelko, 2023), trust in the message source (Vaala et al., 2025), political ideology (Vaala et al., 2022, 2025), and pre-existing beliefs or attitudes (Vraga et al., 2019), consistently emerged as boundary conditions across studies. For example, humor was more effective in low-severity crisis contexts (Xiao & Yu, 2022) and among individuals with lower perceived efficacy, where it increased source liking and subsequently strengthened preventive intentions (D. Wang et al., 2023). Conversely, humor could be less effective or even counterproductive when health risks were perceived as severe or when audiences already possessed high efficacy or strong prior beliefs, as it could dampen risk perceptions or weaken cognitive elaboration.

## 4. Discussion

This systematic review examined how humor functions as a communication strategy when intentionally designed and deployed in social media health communication by professionals or institutions. Drawing on 32 empirical studies, we synthesized evidence on how humor is strategically used in social media health communication, the communication outcomes associated with humor-based messages, and the empirically supported relationships between humor and these outcomes. Overall, the findings indicate that humor has been examined in two primary ways: as a content-level strategy to enhance audience engagement and as a psychological persuasive appeal embedded in health message design and dissemination. Across studies, humor was consistently associated with higher platform-level engagement, such as views, likes, shares, and comments. Beyond engagement, humor directly influenced health message-related affective responses, attitudes, cognitions, and perceptions, which frequently served as proximal outcomes and as mediating mechanisms shaping downstream behavioral intentions. Context and audience characteristics emerged as moderating factors that shaped the effectiveness of humor on communication outcomes.

### 4.1. Effectiveness of Humor in Social Media Health Communication

More than half of the included studies (n = 19, 59.38%) were descriptive in nature and primarily examined humor as a content-level communication strategy for audience engagement on social media platforms. This finding is understandable but also notable because, compared with traditional media, social media platforms provide health communicators with timely and low-cost opportunities to trace and evaluate the engagement metrics as indicators of communication effectiveness (Gough et al., 2017). These metrics have been widely recognized as meaningful indicators of public engagement in social media health communication (Ngai et al., 2020, 2022).

The effectiveness of humor as a content-level communication strategy for increasing social media engagement can be explained through several mechanisms, including attention capture (Kim et al., 2021), the positive emotions elicited by humor (T. Wang & Pavelko, 2023), and reduced defensiveness (Martin, 2007), which may encourage audiences to share humorous health messages with family and friends (So et al., 2016). Moreover, the higher levels of views, likes, and shares associated with humor-based health messages may activate platform algorithms that amplify message visibility and circulation (Heimbach & Hinz, 2018), thereby further enhancing social media engagement.

However, engagement outcomes are not uniform across health topics or audiences. Studies focusing on sensitive health issues, such as sexual health and mental health, suggested that humor-based strategies did not always translate into explicit platform-level engagement (Byron et al., 2013; Yap et al., 2019). One possible explanation is that some audiences may feel embarrassed or uncomfortable publicly “liking” or “sharing” posts related to stigmatized topics such as sexual health or depression. Moreover, engagement outcomes depend on audience preferences and message appeal. When humorous health messages were perceived as unappealing, inappropriate, or mismatched with audience expectations, engagement levels might be correspondingly lower (Kessel et al., 2016; Paek et al., 2013). Together, these findings suggest that while humor can be an effective engagement strategy, its success is closely related to topic sensitivity, message appeal, and audience norms.

The remaining 13 included studies (40.63%) employed experimental designs to examine humor as a psychological persuasive appeal in social media health message design and dissemination. Rather than focusing on platform-level engagement, these studies unpacked the underlying psychological processes through which humor-based health messages influence communication effectiveness. The findings from these experimental studies align closely with established persuasion theories, particularly the Elaboration Likelihood Model (ELM; Petty & Cacioppo, 1986), which explains how individuals process persuasive information through either the peripheral route (low effort, relies on superficial cues like attractiveness, leads to temporary attitudinal change) or the central route (high effort, logic-based, leads to lasting change).

From an ELM perspective, humor can serve as a peripheral cue that enhances source likability and message attractiveness (e.g., T. Wang & Pavelko, 2023; Xiao & Yu, 2022), thereby influencing attitudes when audiences are less motivated or less engaged in effortful cognitive elaboration. At the same time, humor may also facilitate central-route processing by increasing attention and processing fluency (e.g., Kim et al., 2021; T. Wang & Pavelko, 2024), enabling more elaborative engagement with health information when motivation and cognitive resources are sufficient. These findings suggest that humor does not operate solely as a superficial cue but can also affect more effortful cognitive processes when audiences are sufficiently motivated.

### 4.2. Limitations and Challenges of Using Humor in Social Media Health Communication

#### 4.2.1. Potential for Humor to Undermine Message Seriousness

Although humor has been shown to facilitate certain positive communication outcomes, it can also function as a double-edged sword in social media health communication by potentially undermining the seriousness of health messages. Humor may fail to achieve desired outcomes when it lowers perceived risk (Xiao & Yu, 2022), reduces perceived credibility of health information (Kim et al., 2021; Vraga et al., 2019), or weakens the perceived argument strength (Vaala et al., 2025). In particular, when health messages require careful cognitive processing (e.g., correcting misinformation), humor may lead audiences to focus on the entertaining aspects of the message rather than engage in critical evaluation (Vraga et al., 2019).

These findings align with prior concerns that humor can trivialize health threats when the message tone is perceived as incongruent with the seriousness of the issue (Oh et al., 2023; Paek et al., 2013). In this sense, humor has the risk of functioning as a mechanism of epistemic dilution: the entertainment frame becomes primary, while the epistemic content (e.g., what is true and what to do in health-related contexts) is backgrounded or dismissed (Miller et al., 2021). Humor framing can blur the distinction between serious information and entertainment. As a result, audiences may interpret humorous health messages less seriously or perceive them as less authoritative, potentially weakening the persuasive impact of the message (Oh et al., 2023). This potential to undermine message seriousness helps explain the hesitancy observed among health communicators in adopting humor-based approaches (Galea et al., 2025).

In addition, the ironic and satirical tones often embedded in humorous health messages on social media may reduce the perceived responsibility of communicators. When humorous health messages contain offensive or misleading elements (Reijnen et al., 2024), communicators may distance themselves from the message by claiming that it was intended as a joke. Such ambiguity can further weaken the perceived seriousness of the message and make it more difficult for audiences to interpret the communicator’s true intention, which may be associated with disengagement from the information (D’Errico et al., 2024; Paciello et al., 2023). Taken together, these dynamics suggest that humor can dilute message seriousness, blur information intent, and weaken perceived credibility, highlighting the importance of using humor strategically and balancing it with a sufficient level of seriousness, particularly when addressing sensitive or high-stakes health topics, to ensure that the use of humor do not come at the expense of credibility, risk perception, or protective action.

#### 4.2.2. Limited Effectiveness of Humor in Influencing Health Actions

Despite the supportive finding that humorous health messages on social media tend to elicit higher levels of audience engagement, the reviewed evidence suggested that the effectiveness of humor in influencing health-related actions was limited. O’Kane et al.’s (2022) focus group findings revealed that humor alone might be insufficient to convey clear and actionable health information. Social media users preferred humorous posts that were paired with opportunities to access additional information, such as through swipe-through content, suggesting that humor might function effectively as an entry point rather than a standalone persuasive strategy. This limitation aligns with the peripheral route of the ELM (Petty & Cacioppo, 1986), which suggests that when humor functions primarily as a superficially attractive cue, audiences may engage in low-effort processing driven by amusement rather than substantive message elaboration. As a result, humor-induced engagement or momentary attention does not necessarily translate into meaningful cognitive evaluations or sustained behavioral intentions (Walter et al., 2018).

Moreover, among the experimental studies examining the effectiveness of humorous health messages, most focused on how humor shaped health-related attitudes and perceptions, whereas only a small number explicitly tested the psychological mechanisms, such as emotional responses or source liking, that mediate the relationship between humor-based strategies and behavioral intentions (e.g., Seo et al., 2025; T. Wang & Pavelko, 2023, 2024). One possible explanation for this imbalance is that humor’s influence on observable actions may be less immediate or less pronounced than its effects on attitudes and perceptions (Walter et al., 2018), making behavioral outcomes more difficult to detect experimentally. Researchers also acknowledged that interactions on social media or self-reported behavioral intentions do not necessarily translate into actual health behaviors (Apers et al., 2025; Harper et al., 2024), further complicating empirical assessments of humor’s behavioral impact. As a result, research examining how humor influences health behavioral intentions remains limited, and the effectiveness of humor in shaping real-world health actions is still underexplored.

#### 4.2.3. Impracticality of Adopting a Standardized Humor Strategy

The identification of contextual and audience characteristics as moderating factors highlights a fundamental challenge in humor-based social media health communication: its effectiveness varies substantially across platforms, topics, and audiences, making a standardized humor strategy impractical (Galea et al., 2025; Miller et al., 2021). For example, Lee and Chen (2017) found that platform-specific affordances could shape how humor was received. They reported that humorous antitobacco videos generated more favorable communication outcomes on Facebook when embedded in health-focused commenting environments than when circulated on YouTube, suggesting that the same humorous content may perform differently across platforms.

In addition to platform affordances and commenting environments, other contextual and audience characteristics have been found to shape the effectiveness of humor in social media health communication. These include crisis severity (Xiao & Yu, 2022), health topic sensitivity (Lee & Chen, 2017), perceived efficacy (T. Wang & Pavelko, 2023), trust in the message source (Vaala et al., 2025), political ideology (Vaala et al., 2022, 2025), and pre-existing beliefs or attitudes (Vraga et al., 2019). For example, perceived severity of health risks and perceived efficacy play important roles in moderating humor effectiveness. Humor may be less effective when health risks are perceived as highly severe or when audiences already possess high levels of efficacy. These findings are consistent with Limbu et al.’s (2026) review on humor appeals in health communication, which reports that audience characteristics (e.g., age, gender, identity) and the severity of health conditions can influence the effectiveness of humor-based health messages. Specifically, this review indicates that humor tends to be less effective in health messages targeting children, adolescents, and parents and in perceived high-risk health conditions. Together, these studies highlight important boundary conditions for humor effectiveness in health communication and underscore the impracticality of adopting a standardized humor strategy.

Moreover, humor is inherently subjective and audience dependent (Hendriks & Janssen, 2018; Schumacher, 2019), which further complicates the design of universally effective humorous health messages. The success of humor-based health promotion and prevention relies heavily on sufficient understanding of the target audience. Only when humor is perceived as appropriate and acceptable are audiences likely to engage in deeper cognitive processing of health information, which can subsequently influence attitudes and behaviors (Eisend, 2009; S. Ford et al., 2021). Rather than adopting a one-size-fits-all approach, health communicators should prioritize context-sensitive and audience-centered humor strategies. Pretesting humorous messages to assess the audience’s subjective experience and acceptance is therefore essential before large-scale dissemination. Such an approach can help mitigate the risks associated with misaligned humor and enhance the likelihood that humor contributes meaningfully to health communication goals.

#### 4.2.4. Ethical Concerns and Cultural Sensitivity in Humor-Based Health Communication

Designing and disseminating humor-based health messages on social media raises important ethical and cultural challenges. Humor must be employed cautiously to avoid reinforcing stigma, causing offense, or marginalizing specific groups (Hyder, 2022; Lion & Dhaenens, 2023). For example, Reijnen et al. (2024) found that healthy food promotion messages containing jokes that used the word “fat” reduced individuals’ intentions to purchase healthy food. This finding highlights how seemingly lighthearted humor can inadvertently perpetuate stigma and undermine health promotion goals. Accordingly, rhetorical choices in humorous health messages require careful consideration to ensure that they do not stigmatize or demean particular populations.

Poorly designed humor-based messages may also contribute to stigmatization and discrimination, especially when disparagement humor is used (Ferguson & Ford, 2008; T. E. Ford & Ferguson, 2004; Zillmann, 1983). Some audiences may identify with the target of the humor based on characteristics such as gender, race, or health status, leading them to feel personally attacked or excluded (Seo et al., 2025). From the perspective of superiority theory, humor can derive from a sense of dominance over others, positioning the object of humor as inferior (Bardon, 2005; Lintott, 2016). As such, health communicators and researchers should be particularly cautious when humor involves specific social groups, avoiding the construction of “negative exemplars” that reinforce hierarchies or social exclusion.

Cultural sensitivity is equally critical in the application of humor-based health communication strategies on social media. Humor is highly culture-dependent (J. G. Lu, 2023), and content that resonates positively in one cultural context may be inappropriate or ineffective in another. For instance, Harper et al. (2024) reported that the use of a Christian holiday in the “DryByChristmas” pelvic floor muscle training campaign limited its suitability for cross-cultural adaptation. More broadly, cultural miscalculations in humor-based health messaging can provoke public backlash, damage institutional credibility, and erode trust in health communicators (Galea et al., 2025). These risks help explain the reluctance of some health professionals and organizations to adopt humor-based strategies, as avoiding humor is perceived as a way to minimize ethical and reputational risks (Galea et al., 2025).

However, completely avoiding humor also entails forfeiting its potential communication benefits. Rather than abandoning humor altogether, health communicators should engage in systematic pretesting to assess audience interpretations, ethical acceptability, and cultural appropriateness. By evaluating humor acceptance among target populations or even with the broader public when appropriate, humor can be incorporated in ways that are respectful, inclusive, and aligned with public health goals (Galea et al., 2025; Miller et al., 2021; Verma, 2022), while minimizing ethical and cultural risks at the message design stage.

### 4.3. Future Directions of Humor in Social Media Health Communication

#### 4.3.1. Advancing Mechanism Research Through Behavioral Frameworks

Future research should move beyond identifying whether humor is effective to more systematically examining when, how, and for whom humor operates in social media health communication. The conceptual framework proposed in this review offers a foundation that can be further tested and refined using established behavioral frameworks, particularly the Theoretical Domains Framework (TDF; Cane et al., 2012). The TDF integrates 33 behavioral theories into 14 domains, including the domain of “‘Knowledge,’ ‘Skills,’ ‘Social/Professional Role and Identity,’ ‘Beliefs about Capabilities,’ ‘Optimism,’ ‘Beliefs about Consequences,’ ‘Reinforcement,’ ‘Intentions,’ ‘Goals,’ ‘Memory, Attention and Decision Processes,’ ‘Environmental Context and Resources,’ ‘Social Influences,’ ‘Emotions,’ and ‘Behavioral Regulation’” (Cane et al., 2012), and these domains can guide the design and evaluation of humor-based behavior change interventions.

The studies included in this review have already examined several TDF-relevant constructs as mechanisms through which humor operates. For instance, perceived self-efficacy in the Belief about Capabilities domain was shown to condition humor’s persuasive effects (T. Wang & Pavelko, 2023), attention played a mediating role in misinformation correction (Kim et al., 2021), and emotional responses such as fear and guilt shaped risk perceptions and behavioral intentions (Alhabash et al., 2022; Vaala et al., 2022). However, many TDF domains remain underexplored in the context of humor-based social media health communication.

One notable gap lies within the Memory, Attention, and Decision Processes domain. Although humor has long been theorized to enhance memory retention and recall (Cline & Kellaris, 2007), empirical evidence testing this assumption in social media health communication is largely absent. Future studies could investigate whether humorous framing improves long-term recall of health information or facilitates decision-making under information overload conditions characteristic of social media environments. Advanced methodological approaches, such as neuroimaging techniques (e.g., Functional Magnetic Resonance Imaging and Electroencephalography), could help capture neural and cognitive markers of attention, memory encoding, and retrieval associated with humorous health messages.

Another promising direction concerns the Social Influences domain of the TDF. Existing studies have primarily focused on individual-level outcomes, such as attitudes, perceptions, and behavioral intentions. However, social media is inherently social, and humor-based health messages designed by professionals may exert influence through mechanisms such as perceived social norms, peer endorsement, social support, and group conformity. Future research should examine how humor interacts with these social processes in experimental designs. For example, whether humorous health messages are more likely to be shared within peer networks (Byron et al., 2013; Evers et al., 2013), normalize health behaviors, or reduce social stigma through collective engagement, integrating social influence mechanisms into humor-based models would offer a more comprehensive understanding of how humor functions within networked digital environments.

#### 4.3.2. Understanding the Use of Humor Through Cultural Dimensions

Given the importance of cultural sensitivity in humor-based social media health communication, future research should further examine how humor operates across diverse cultural contexts. Prior research indicates that humor perception is culturally contingent. For example, J. G. Lu (2023) found that individuals from different cultural backgrounds interpret and evaluate humor differently, with North Americans generally perceiving humor more positively than East Asians. Such findings underscore the need to move beyond culturally homogeneous samples when examining humor’s persuasive effects in health communication.

Hofstede’s Cultural Dimensions Theory (Hofstede, 2011) offers a useful framework for understanding cross-cultural variation in responses to humorous health messaging. Cultural dimensions such as individualism–collectivism, uncertainty avoidance, power distance, and indulgence-restraint may shape audiences’ acceptance of humor, perceptions of appropriateness, and emotional and cognitive responses to humor-based health messages. For instance, ironic humor may resonate more strongly in individualistic and indulgent cultures than in collectivist and restrained cultures. By systematically examining how cultural values interact with humorous framing in social media health messaging, future studies can clarify why humor facilitates engagement and persuasive outcomes in some cultural settings while eliciting trivialization or resistance in others, thereby contributing to more culturally sensitive and effective health communication strategies.

#### 4.3.3. Leveraging Social Media User Comments to Examine Humor Effects

In addition to establishing quantitative associations between humorous framing and social media engagement, future studies could further investigate how humor strategies shape users’ responses by systematically examining the commenting data under humor-based health promotion posts. Among the included studies, only one (Kreindler et al., 2024) examined the effects of humor on communication outcomes through a qualitative analysis of user comments on health promotion messages. The findings were mixed, with some commenters praising the humor-based strategy, while others expressed disapproval of trivializing serious health topics, describing the message as “distinguishingly childish.” Compared with traditional media channels such as television advertisements or radio programs, social media platforms offer a unique environment in which large-scale, unsolicited, and publicly visible audience feedback can be directly observed. Analyzing the valence and content of user comments allows researchers to move beyond engagement metrics to assess how audiences interpret and respond to humorous health messages (Ngai et al., 2022; Paek et al., 2013). For example, computational approaches such as topic modeling and sentiment analysis can be employed to identify dominant themes and emotional valence (S. Chen et al., 2025) within comment threads. Content analysis can provide deeper insights into how humor facilitates positive health-related attitudes, health knowledge enhancement, misperception corrections, information sharing, positive health behavior implementation, or trivializes health risks. Together, these approaches to commenting on data can help explore and clarify the communicative mechanisms through which humor enhances or undermines the effectiveness of health communication on social media.

#### 4.3.4. Conducting Longitudinal Studies to Understand Humor Effects

All included studies in the current review are cross-sectional in nature, which limits understanding of the temporal dynamics of humor-based social media health communication. Future research could adopt longitudinal designs to examine whether the effects of humorous strategies in social media health communication persist, increase, or diminish over time, particularly in shaping individuals’ health-related attitudes, perceptions, and behaviors. Building on Nabi (2016), who argues that humorous messages may generate sleeper effects (a delayed persuasive impact that strengthens after initial exposure), longitudinal studies can test whether such delayed effects emerge within social media environments. Unlike traditional media, social media platforms enable repeated exposure through algorithmic recirculation, reposting, and echo chamber dynamics, which may continuously reinforce similar messages based on users’ browsing histories. Future studies could examine whether humorous health messages facilitate sustained persuasion, delayed attitude change, or gradual behavioral adoption over time, thereby offering a more comprehensive understanding of the long-term effectiveness of humor in digital health communication contexts.

### 4.4. Limitations of the Current Study

Like all research, this systematic review has several limitations that should be acknowledged. First, the review focused exclusively on humor-based health communication on social media designed and disseminated by healthcare professionals, health organizations, and researchers. While this focus aligns with the study’s aim of examining humor as an intentional communication strategy, it excludes potentially meaningful uses of humor generated by lay users, such as humor employed as a coping strategy during health crises or everyday health discussions. Future research could broaden the analytical scope by including humor created or shared by other actors, such as lay users, influencers, or celebrities, to better capture the diverse ways humor functions in social media health communication contexts.

Second, the studies included in this review demonstrate substantial contextual variation. The context-dependent nature of humor makes it difficult to draw universal conclusions about its effectiveness in social media health communication, particularly when humor is used as a persuasive appeal. Future research should further investigate humor effectiveness across specific contexts, such as particular health conditions, audience groups, or cultural settings, to better understand the mechanisms through which humor enhances or undermines communication outcomes.

Third, the reviewed studies employed diverse methodological approaches, including experiments, surveys, content analysis, observational studies, interviews, and focus groups. While this methodological diversity provides a rich body of evidence, it also introduces challenges for synthesis, as different designs conceptualize and measure humor and communication outcomes in different ways. Given this heterogeneity, this review adopted a narrative synthesis to integrate findings across studies. Future research could benefit from developing more standardized conceptualizations and measurements of humor-based strategies and communication outcomes. In addition, as the body of experimental research continues to grow, meta-analytic approaches may become feasible for quantitatively synthesizing the effects of humor in health communication, thereby facilitating more systematic evidence integration.

Finally, this review included only peer-reviewed journal articles published in English, thereby excluding grey literature such as conference proceedings, dissertations, reports, and non-English-language publications. As a result, emerging research or culturally specific insights that have not yet appeared in indexed journals may have been overlooked. Future reviews could incorporate grey literature and studies published in other languages to provide a more comprehensive and globally representative understanding of how humor operates in social media health communication across different cultural and research contexts.

## 5. Conclusions

This study is one of the earliest reviews exploring how humor functions as a communication strategy in professional health communication on social media. Synthesizing evidence from 32 empirical studies, this review demonstrates that humor effectively enhances audience engagement and shapes affective, cognitive, and perceptual responses, which in turn influence health-related attitudes and behavioral intentions. Importantly, the effectiveness of humor is conditional, varying across health contexts and audience characteristics. By integrating fragmented findings, this review proposes a conceptual framework that clarifies the pathways through which humor operates from message design to psychological mechanisms and communication outcomes, offering theoretical coherence and practical guidance for health message designers, communicators, policymakers, health organizations, and researchers. At the same time, this review highlights key challenges, including the risk of trivializing serious health issues, limited direct effects of humor in influencing behavior change, the impracticality of adopting standardized humor strategies, and the ethical and cultural sensitivities of humor. Future research should further test and refine this framework by examining humor through established behavioral theories, exploring cross-cultural dynamics, incorporating social interaction processes by examining comments of humor-based posts, and employing longitudinal designs to have a more comprehensive understanding of humor’s communication effectiveness in social media health communication. Overall, this review positions humor as a promising yet nuanced strategy in digital health communication, with substantial potential for vast research possibilities and applied impact.

## Data Availability

All data generated or analyzed during this study are included in this published article.

## Appendix A

**Table A1:** Quality assessment of included studies using the Mixed Methods Appraisal Tool.

Reference	All Studies		Qualitative Studies					Quantitative Nonrandomized					Quantitative Descriptive					Mixed Methods				
	S1	S2	1.1	1.2	1.3	1.4	1.5	3.1	3.2	3.3	3.4	3.5	4.1	4.2	4.3	4.4	4.5	5.1	5.2	5.3	5.4	5.5
(Al-Rawi & Zemenchik, 2023)	Y	Y	Y	Y	Y	Y	Y															
(Alhabash et al., 2022)	Y	Y						Y	Y	Y	Y	Y										
(Apers et al., 2025)	Y	Y						Y	Y	Y	Y	Y										
(Bian et al., 2021)	Y	Y											Y	Y	Y	Y	Y					
(Byron et al., 2013)	Y	Y	Y	Y	Y	Y	Y															
(A. Chen et al., 2025)	Y	Y	Y	Y	Y	Y	Y															
(Droog et al., 2025)	Y	Y						Y	Y	Y	Y	Y										
(Evers et al., 2013)	Y	Y	Y	Y	Y	Y	Y															
(Galea et al., 2025)	Y	Y	Y	Y	Y	Y	Y															
(Gough et al., 2017)	Y	Y																Y	Y	Y	C	Y
(Harper et al., 2024)	Y	Y											Y	Y	Y	C	Y					
(Kim et al., 2021)	Y	Y						Y	Y	Y	Y	Y										
(Kreindler et al., 2024)	Y	Y																Y	Y	Y	Y	Y
(Lee & Chen, 2017)	Y	Y						Y	Y	Y	N	Y										
(Lister et al., 2015)	Y	Y											Y	Y	Y	C	Y					
(Love et al., 2026)	Y	Y											C	C	Y	C	Y					
(O’Kane et al., 2022)	Y	Y																Y	Y	Y	C	Y
(Pfeiffer et al., 2014)	Y	Y																Y	Y	Y	C	Y
(Reijnen et al., 2024)	Y	Y						Y	Y	Y	Y	Y										
(Ruco et al., 2022)	Y	Y	Y	Y	Y	Y	Y															
(Seo et al., 2025)	Y	Y						Y	Y	Y	Y	Y										
(Teoh et al., 2019)	Y	Y											Y	Y	Y	Y	Y					
(Vaala et al., 2022)	Y	Y						Y	Y	Y	Y	Y										
(Vaala et al., 2025)	Y	Y						Y	Y	Y	Y	Y										
(Vraga et al., 2019)	Y	Y						Y	Y	Y	Y	Y										
(D. Wang et al., 2023)	Y	Y											C	Y	Y	C	Y					
(T. Wang & Pavelko, 2023)	Y	Y						Y	Y	Y	Y	Y										
(T. Wang & Pavelko, 2024)	Y	Y						Y	Y	Y	Y	Y										
(Weeks et al., 2022)	Y	Y																Y	Y	Y	C	Y
(Xiao & Yu, 2022)	Y	Y						Y	Y	Y	Y	Y										
(Yap et al., 2019)	Y	Y											Y	Y	Y	C	Y					
(Zhang et al., 2025)	Y	Y											Y	Y	Y	C	Y					

Note. Y = Yes, N = No, C = Can’t tell, S1: Are there clear research questions? S2. Do the collected data allow us to address the research questions? 1.1. Is the qualitative approach appropriate to answer the research question? 1.2. Are the qualitative data collection methods adequate to address the research question? 1.3. Are the findings adequately derived from the data? 1.4. Is the interpretation of results sufficiently substantiated by data? 1.5. Is there coherence between qualitative data sources, collection, analysis and interpretation? 3.1. Are the participants representative of the target population? 3.2. Are measurements appropriate regarding both the outcome and intervention (or exposure)? 3.3. Are there complete outcome data? 3.4. Are the confounders accounted for in the design and analysis? 3.5. During the study period, was the intervention administered (or exposure occurred) as intended? 4. Quantitative descriptive 4.1. Is the sampling strategy relevant to address the research question? 4.2. Is the sample representative of the target population? 4.3. Are the measurements appropriate? 4.4. Is the risk of nonresponse bias low? 4.5. Is the statistical analysis appropriate to answer the research question? 5.1. Is there an adequate rationale for using a mixed methods design to address the research question? 5.2. Are the different components of the study effectively integrated to answer the research question? 5.3. Are the outputs of the integration of qualitative and quantitative components adequately interpreted? 5.4. Are divergences and inconsistencies between quantitative and qualitative results adequately addressed? 5.5. Do the different components of the study adhere to the quality criteria of each tradition of the methods involved?

## Appendix B

**Table A2:** Data extraction table.

Reference	Study Setting and Research Aims	Country	Focused Social Media Platforms	Research Methods	Samples/Participants	Humor’s Role as a Communication Strategy	Communication Outcome(s) of Using Humor-Based Strategies	The Relationships Between Humor and Communication Outcomes
(Al-Rawi & Zemenchik, 2023)	This study analyzed 2166 TikTok videos created by healthcare workers about COVID-19 to understand audience engagement with dominant themes.	Not mentioned	TikTok	Content analysis	2166 healthcare workers created TikTok videos about COVID-19.	Humor as a communication strategy for audience engagement.	Audience engagement metrics: shares, hearts, play counts, and comments.	Humor/satire videos generated higher audience engagement. Specifically, humor/satire theme had the highest shares and high hearts/plays compared with other themes.
(Alhabash et al., 2022)	This study examined and compared the effects of humor-based versus fear-based persuasive appeals in social media-oriented public service announcements (PSAs) on individuals’ intentions to purchase medications through social media.	United States	Multiple social media platforms, such as Facebook, Instagram, Twitter, etc.	Online experiment	730 social media users recruited online in the United States, mean age 38.44, 65.07% male.	Humor as a persuasive appeal for health prevention, discouraging unsafe medication purchasing through social media.	Psychological reactance (threat to freedom, anger); attitudes toward the PSA; viral behavioral intentions (VBIs, liking, sharing, commenting); intentions to purchase medications via social media.	Humor appeals were associated with lower perceived threat to freedom, reduced anger, and more favorable as toward the PSA; Humor appeals also produced marginally lower purchase intentions and lower VBIs than fear appeals; Pre-existing risk perception moderated the effects of appeal type on perceived threat to freedom, attitudes, and VBI; Gender, age, education, income, prescription use, insurance status, and household income were included as control variables.
(Apers et al., 2025)	This study evaluated whether humor-based (vs. narrative- and norm-based) social media strategies increase intention to seek additional radon-related information and intention to test for radon in Radon Buster public health campaign. It also examined how the effects of social media ads varied across Austria, Belgium, Ireland, and Slovenia.	Austria, Belgium, Ireland, Slovenia.	Facebook	Online experiment	1677 adults who were homeowners and lived in radon-risk areas in Austria, Belgium, Ireland, or Slovenia, mean age 51.88, 50.6% male.	Humor as a persuasive appeal for health promotion, encouraging radon-related information-seeking.	Intention to seek additional information about radon.	Humor-based Facebook ads significantly increased intention to seek additional information; advertisement appreciation, age, sex, prior exposure to radon communication were included as control variables.
(Bian et al., 2021)	This study examined how specific content features, including degree of humor, influenced health promotion outcomes through a hospital-run WeChat Official Account (WOA, run by the Shanghai Ruijin Hospital).	China	WeChat	Content analysis	103 WeChat health articles from Shanghai Ruijin Hospital’s WOA.	Humor as a communication strategy for audience engagement.	Influence index (i.e., standardized views, reads, shares); audience engagement metrics (likes, shares, reads).	Higher degree of humor positively predicted article influence; articles using humor and caricatures were more likely to receive higher engagement, including likes and dissemination reach.
(Byron et al., 2013)	This study explored how young people in Sydney and New South Wales perceived the potential and suitability for sexual health promotion via social media such as Facebook, YouTube, and Twitter.	Australia	Facebook, YouTube, Twitter.	Focus groups	22 Australian young people, aged 16–22.	Humor as a communication strategy for audience engagement.	Perceived acceptability of sexual health content in social media; willingness to engage with sexual health messages; message retention.	Humor was perceived to increase message acceptability and shareability by allowing users to engage with sexual health content without implicating their identity or sexual status; humorous videos were seen as more memorable and less stigmatizing, facilitating peer sharing, whereas “liking” a sexual health Facebook page might be embarrassing.
(A. Chen et al., 2025)	This study conducted a qualitative study to inform the development of the Social Media and Sexual Health campaign and aimed at promoting HIV pre-exposure prophylaxis (PrEP) uptake among young Black and Latino men who have sex with men (YBLMSM) through social media influencers and short videos.	United States	Multiple social media platforms, such as Instagram (primary), Facebook, Twitter (X), TikTok, YouTube, and Snapchat.	Focus groups	22 YBLMSM, aged 18–29.	Humor as a communication strategy for audience engagement.	Perceived engagement with PrEP messages; willingness to attend to and share content; perceived credibility and seriousness of PrEP Information.	Humor was perceived to increase engagement and sharing, but it might also undermine message seriousness; effective PrEP communication required a balance between humor and seriousness.
(Droog et al., 2025)	This study examined whether satirical humor was an effective correction strategy to counter misinformation about contraception on social media and whether it outperformed non-humorous corrections.	Netherlands	TikTok & YouTube	Online experiment	678 Dutch-speaking women aged 18–35 who had high contraceptive use and were actively engaged with social media.	Humor as a persuasive appeal for misinformation correction.	Misinformation believability; contraception beliefs; attitudes toward natural and hormonal contraception.	Both satirical and non-humorous corrections reduced belief in false claims and reduced favorable attitudes toward natural contraception compared with no correction; satirical correction also reduced perceived believability of misinformation. However, humor correction did not increase belief in true claims or consistently improve attitudes toward hormonal contraception. Humor was effective for debunking false beliefs but limited for fostering acceptance of accurate information.
(Evers et al., 2013)	This study explored how young people in Sydney and New South Wales perceived the potential and suitability for sexual health promotion via social media such as Facebook, YouTube, and Twitter.	Australia	Multiple social media platforms, such as YouTube (primary), Facebook, Twitter.	Focus groups	22 Australian young people aged 16–22.	Humor as a communication strategy for audience engagement.	Perceived acceptability of sexual health content in social media; willingness to engage with sexual health messages; addressing stigma concerns of sexual health communication.	Humor was perceived to increase message acceptability and shareability by allowing users to engage with sexual health content without implicating their identity or sexual status; humorous content reduced stigmatization of sexual health and facilitated peer sharing.
(Galea et al., 2025)	This study examined how a large public health organisation (Queensland Health) used social media to communicate with the public, exploring the use of humor in social media health communication.	Australia	Not mentioned	Interviews	19 Queensland Health social media administrators and communication professionals.	Humor as a communication strategy for audience engagement.	Perceived audience engagement; accessibility and comprehension of health information; organizational trust and reputation; perceived health literacy improvement.	Humor was perceived to enhance engagement, relatability, and accessibility of health messages and serve as an effective entry point for sensitive topics, particularly for younger audiences; misjudged humor could undermine message seriousness, trigger cultural misinterpretation, damage trust, and provoke backlash, leading organizations to adopt a cautious or selective use of humor.
(Gough et al., 2017)	This study tested the feasibility of a public health social media campaign focused on skin cancer prevention, using different message frames (humor, shock/disgust, informative, personal stories, opportunistic). It evaluated how different message frames influenced audience engagement.	United Kingdom	Twitter	Quasi-experimental study	Tweets as campaign messages exposed to users. The number of Tweets was not specified.	Humor as a communication strategy for audience engagement.	Audience engagement metrics: Twitter analytics data including impressions, engagements, engagement rate, retweets.	Humorous messages generated the highest engagement rate and engagement counts among all message frames.
(Harper et al., 2024)	This patient and public involvement study examined how women engaged with a humorous social media campaign (#DryByChristmas) promoting pelvic floor muscle training (PFMT), informing the design of a digital behavioral intervention.	United Kingdom	Instagram	Observational study	Instagram posts for the #DryByChristmas campaign. The number of posts was not specified.	Humor as a communication strategy for audience engagement.	Audience engagement metrics: reach, likes, comments, profile visits, follower growth.	Humorous digital nudges were associated with higher levels of engagement, particularly likes and profile visits; engagement was strongest for content targeting opportunity and motivation in behavioral change promotion for PFMT.
(Kim et al., 2021)	This study compared humor-based versus logic-based correction strategies in correcting HPV vaccine misinformation and examined attention and message credibility as mediating mechanisms in reducing misperceptions.	United States	Twitter	Laboratory-based experiment	61 university students, mean age 20, 37.7% male.	Humor as a persuasive appeal for misinformation correction.	Visual attention based on eye-tracking data (total fixation duration to text/image area of interests); message credibility (misinformation tweet credibility; correction tweet credibility); HPV vaccine misperceptions.	Humor increased attention to the correction image and to misinformation text, and increased attention to the humorous correction image indirectly reduced HPV misperceptions by lowering misinformation credibility. Pretest HPV misperceptions were included as the control variable.
(Kreindler et al., 2024)	This study evaluated whether a research-based musical satire (Larry Saves the Canadian Healthcare System) could increase public understanding, interest, and capacity to engage with complex health policy issues related to emergency department crowding.	Canada	YouTube	Mixed-methods	The Larry videos on social media platforms and 117 comments under these videos; 92 university students, 45% under 25, 23% male.	Humor as a communication strategy for audience engagement, using musical comedy to make complex system-level health policy issues accessible, memorable, and engaging.	Audience engagement metrics: views, likes, dislikes, comments, and shares; perceived informativeness, memorability, enjoyment; self-reported increases in knowledge, interest, confidence to discuss health policy; intention to share.	Satirical humor was associated with high engagement and positive reception, with viewers frequently praising the video’s humor in comments. Student audiences reported increased knowledge, interest, and confidence to engage in health policy discussions. However, some viewers perceived risks of trivialization of serious health topics.
(Lee & Chen, 2017)	This study investigated the effectiveness of humorous antitobacco videos varies by social media platform (Facebook vs. YouTube) and circulating context (health-focused vs. humor-focused.	United States	Facebook & YouTube	Online experiment	213 nonsmoking college students, mean age 20, 32% male.	Humor as a persuasive appeal for health prevention (smoking prevention).	Risk perception of smoking; attitudes toward smokers; intentions to avoid smoking.	Humorous antitobacco videos produced stronger prevention outcomes when circulated on Facebook in a health-focused commenting environment, resulting in higher risk perception of smoking, less positive attitudes toward smokers, and greater intention to avoid smoking.
(Lister et al., 2015)	This study examined the effectiveness of the Laugh Model, a social media-based marketing framework emphasizing humor and entertainment, in promoting healthy family meals through a statewide public health campaign.	United States	Facebook & Twitter	Observational study	Social media posts for the promotion of healthy family meals in Utah. The number of posts was not specified.	Humor as a communication strategy for audience engagement	Audience engagement outcomes: impressions, likes, shares, comments, clicks, website visits, engagement rates, cost-effectiveness metrics.	Humorous and entertaining content generated the highest engagement rates, outperforming purely educational posts. Humor facilitated attention, sharing, and traffic to the campaign website, supporting the Laugh Model’s proposition that entertainment-first strategies enhance public engagement and cost-effective reach in public health communication.
(Love et al., 2026)	This study evaluated a multiyear, multipartner creative social media initiative “#GetLeadCheckedTexas.” This campaign was designed to increase awareness of childhood blood lead poisoning and promote blood lead testing among young children.	United States	Not mentioned	Observational study	Parents, healthcare providers, and Texas Department of State Health Services (DSHS) staff involved in formative research; campaign audiences included parents and providers reached via DSHS social media channels.	Humor as a communication strategy for audience engagement.	Audience engagement metrics.	Humorous messages were associated with increased attention and engagement; this was further reflected in substantial increases in website page views and downloads of screening resources.
(O’Kane et al., 2022)	This study evaluated the feasibility and acceptability of using Instagram to engage post-graduate students in a mass-communication social media-based health intervention #WeeStepsToHealth, focusing on physical activity, nutrition, and general wellbeing	United Kingdom	Instagram	Observational study and focus group	Post-graduate students at Queen’s University Belfast, focus group (n = 8), 7 female and 1 male.	Humor as a communication strategy for audience engagement.	Audience engagement metrics: likes, comments, follower growth; attention attraction; message retention.	Humorous posts achieved moderately higher engagement than non-humorous posts (median 10 vs. 8 likes); qualitative data indicated humor increased attention, recall, and willingness to engage with content, but humor alone was insufficient to drive behavior change and needed to be paired with credible information and signposting to trusted sources.
(Pfeiffer et al., 2014)	This study examined adolescents’ use of social media and their perceptions of social media as a platform for sexual and reproductive health (SRH) communication.	Tanzania	Facebook & Twitter	Interviews	8 adolescents, 4 girls and 4 boys.	Humor as a communication strategy for audience engagement.	Perceived acceptability of SRH content; willingness to attend to and engage with SRH messages on social media.	Adolescents perceived humorous and entertaining content as more likely to attract attention and engagement with SRH information, particularly when combined with youth role models like musicians and actors. Humor was also viewed as avoiding social risk and stigma.
(Reijnen et al., 2024)	This study examined whether humorous content embedded in Instagram feeds influenced users’ willingness to purchase healthy food, and tested how joke content and post position shaped purchase intentions of promoted healthy food.	Switzerland	Instagram	Online experiment	411 university students, mean age 24.19, 70.6% female.	Humor as a persuasive appeal for health promotion, which was operationalized as jokes preceding a healthy food post.	Intention to buy healthy food.	Humorous content without the word “fat” received higher level of purchase intention compared with “fat” jokes.
(Ruco et al., 2022)	This study developed and evaluated Facebook messages to promote colorectal cancer (CRC) screening uptake and to identify message characteristics preferred by screen-eligible adults.	Canada	Facebook	Focus groups	45 Facebook users aged 50–74, 44.4% male.	Humor as a communication strategy for audience engagement.	Message acceptability; perceived credibility; attention and recall; willingness to seek information or consider screening.	Humor-based messages produced mixed reactions: some participants found humorous messages attention-grabbing and engaging, but others perceived them as offensive or unprofessional. Overall, humorous content was less consistently preferred than messages with a positive, reassuring, and educational tone. The use of humor was recommended to proceed with caution, as it risked undermining credibility and screening intentions if poorly calibrated.
(Seo et al., 2025)	This study examined how the sequence of humor and risk information in social media PSAs affected persuasion outcomes on vaccination and whether these effects depend on individuals’ vaccination calculation.	United States	Instagram	Online experiment	142 U.S. adults, mean age 50.54, 55.6% male.	Humor as a persuasive appeal for health prevention, discrediting vaccine misinformation.	Perceived vaccine effectiveness; attitude toward COVID-19 vaccination; COVID-19 vaccination intention.	Humor’s effectiveness depends on sequencing and audience psychological antecedents (vaccination calculation in this study). Among individuals with high vaccination calculation, presenting humor first was associated with higher perceived vaccine effectiveness, which in turn led to more positive attitudes and stronger vaccination intentions; Among individuals with low vaccination calculation, presenting humor after risk information was associated with higher perceived vaccine effectiveness.
(Teoh et al., 2019)	This study examined which types of Instagram-style graphics were most appealing for promoting HPV vaccination among young adults, with the goal of improving social media-based health promotion.	United States	Instagram	Survey	1037 young adults recruited at the Minnesota State Fair, mean age 22, 36.1% male.	Humor as a communication strategy for audience engagement, eliciting positive emotional appeal and capturing attention in fast-scrolling social media environments.	Perceived likelihood of stopping to read a post (“stop-and-read” intention).	Humorous graphics were rated significantly more appealing than disease photos and patient photos and comparable to infographics; Humor modestly increased attention-related outcomes, particularly among vaccinated individuals and those with higher eHealth literacy. Gender, race, and age were included as control variables.
(Vaala et al., 2022)	This study examined how different emotional appeals (including humor) and message source (CDC vs. celebrity) influenced audiences’ emotional responses and downstream perceptions of COVID-19 threat and social distancing efficacy, and how these relationships varied by political ideology.	United States	Twitter	Online experiment	415 U.S. citizens, with 242 Democrats and 173 Republicans.	Humor as a persuasive appeal for health prevention, encouraging social distancing.	Emotional responses toward health messages (fear, guilt).	Humor appeals from the CDC elicited lower fear and guilt, which were associated with lower susceptibility to COVID-19, severity of COVID-19.
(Vaala et al., 2025)	This study examined how different emotional appeals (including humor) and message frames in CDC-attributed Twitter messages influence U.S. adults’ emotional, cognitive, and efficacy-related responses to COVID19 risk-reduction behaviors, and how these effects varied by trust in the message source.	United States	Twitter	Online experiment	442 U.S. adult citizens.	Humor as a persuasive appeal for health prevention, encouraging social distancing.	Emotional responses (hope, fear, annoyance); cognitive responses (perceived argument strength).	Humorous tweets reduced fear, but also weakened perceived argument strength, especially among high-trust participants.
(Vraga et al., 2019)	This study tested the effectiveness of humor-based versus logic-based corrective responses to social media misinformation across health, science, and political issues, with a specific focus on HPV vaccination as a health topic.	United States	Twitter	Online experiment	406 U.S. social media users (n = 406), aged 25–34, 54% male.	Humor as a persuasive appeal for misinformation correction.	Misperceptions; credibility of misinformation tweet; credibility of corrective reply tweet.	Humor-based corrections significantly reduced misperceptions for HPV vaccination; Humor-based corrections were rated as more humorous but less credible than logic-based corrections; among participants already convinced of vaccine safety, humor-based corrections helped prevent backfire effects that occurred with logic-based corrections; humor was less effective than logic-based corrections for participants with strong initial misperceptions but more acceptable for supportive audiences. Pre-test attitude was included as a controlled variable.
(D. Wang et al., 2023)	This study conducted pre-post evaluation of a meme-based social media campaign (GetWaivered), aiming at increasing engagement with and enrollment in the web-based Drug Enforcement Administration (DEA)-X waiver training for clinicians to address opioid use disorder.	United States	Twitter, Instagram, Facebook	Observational study	The GetWaivered memes disseminated on Twitter, Instagram, and Facebook. Target audience was clinicians who were eligible to prescribe buprenorphine.	Humor as a communication strategy for audience engagement.	Social media engagement (likes, shares, comments); website traffic (page views, users, sessions); training enrollment and attendance.	Humor-based meme promotion was associated with significant increases in engagement and action; compared with pre-campaign months, the campaign period showed large increases in page views (+7602), new users (+5878), and sessions (+5990), as well as higher training registrations. Findings suggest humor facilitated attention, increased engagement, and enhanced website traffic, supporting memes as an effective driver of professional health education mobilization.
(T. Wang & Pavelko, 2023)	This study examined the effects of incongruity humor (vs. no humor) in COVID-19 prevention messages (hand washing, mask wearing, social distancing) on social media and how perceived efficacy moderated humor effects.	United States	Not mentioned	Online experiment	294 U.S. adults, mean age 40.77, 59.9% male.	Humor as a persuasive appeal for health prevention, encouraging preventive health behaviors during COVID-19, including hand washing, mask wearing, and social distancing.	Source liking; attitudes toward preventive health behaviors; behavioral intentions (hand washing, mask wearing, social distancing).	Humor operated through a moderated serial mediation, whereby the effect of humor on behavioral intentions depended on perceived efficacy and was transmitted sequentially through source liking and attitudes toward preventive health behaviors; among individuals with low perceived efficacy, humor increased source liking, which in turn led to more favorable attitudes and stronger behavioral intentions; among individuals with high perceived efficacy, humor was indirectly associated with less favorable attitudes and weaker behavioral intentions.
(T. Wang & Pavelko, 2024)	This study investigated how different types of humor (incongruity vs. aggressive) interacted with regulator focus framing (promotion vs. prevention) in social media health messages to influence audience engagement intentions.	United States	Not mentioned	Online experiment	Two rounds of studies, with 156 U.S. adults in the first study and 882 U.S. adults in the second study.	Humor as a persuasive appeal for health prevention, encouraging preventive health behaviors during COVID-19, including hand washing, mask wearing, and social distancing.	Social proximity to message source; content endorsement intentions (liking, sharing, recommending health posts); health information engagement intentions (interest in and attention to related health content).	Humor type increased social proximity (Study 1); humor type x regulator focus framing influenced processing fluency, which in turn influenced content endorsement and health information engagement intentions (Study 2).
(Weeks et al., 2022)	This study evaluated a year-long COVID-19 social media campaign designed to identify message characteristics associated with higher audience reach and engagement among American Indian and Alaska Native communities.	United States	Twitter	Mixed methods	162 Twitter messages from Center for American Indian Health Twitter account generated 425,834 impressions and 6016 engagements; 10 key-informant interviews with Tribal Advisory Board members.	Humor as a communication strategy for audience engagement.	Audience engagement outcomes: impressions (reach), engagements (the number of user interactions), engagement rate (the number of engagements a tweet has per impression).	Humor-themed posts were significantly more likely to generate high engagement rates.
(Xiao & Yu, 2022)	This study examined the effectiveness of humorous versus non-humorous message framing in communicating social distancing during the early phase of COVID-19 on social media, and testing how crisis severity phase (low vs. high) moderated the effects of humor.	China	Douyin (TikTok China)	Online experiment	139 users of Douyin, mean age 27, 48% male.	Humor as a persuasive appeal for health prevention, encouraging social distancing.	Online engagement intentions (liking, sharing, commenting); offline engagement intentions (intended compliance with social distancing); source likability; perceived risk of COVID-19.	Crisis severity moderated the effects of humor on online engagement intentions, offline engagement intentions, source likability, and perceived COVID-19 risk. Specifically, humor was more likely to enhance engagement intentions and source likability under low-severity conditions, whereas its effectiveness diminished when the crisis was perceived as highly severe.
(Yap et al., 2019)	This study examined the types of message appeals used in mental health communication on social media by Australian mental health organizations.	Australia	YouTube	Content analysis	65 YouTube videos on depression, anxiety, and stigma reduction produced by Australian mental health organizations.	Humor as a communication strategy for audience engagement.	Audience engagement metrics: number of likes, comments, and shares.	Humor appeals were negatively associated with the number of likes and showed no significant association with comments or shares, compared with other appeals. Humor was less effective in driving engagement.
(Zhang et al., 2025)	This study examined how Chinese celebrity physicians on short-video platforms used different content, emotional, and format strategies to enhance audience engagement.	China	Douyin (TikTok China)	Content analysis	240 top-ranked short videos on Douyin from 30 celebrity physicians.	Humor as a communication strategy for audience engagement.	Audience engagement metrics: likes, saves, shares, comments.	Humorous short videos generated higher engagement levels than many other emotional types, such as hopeful, educational, and excitement, particularly increasing likes and saves. Humor did not substantially increase deeper interactions, such as comments and shares.

Note. Extracted data was related to humor-based social media health communication. If included studies employed more than one study in one article using different research methods, only the humor-related study was extracted and analyzed.
